# Blue Light added with Red LEDs Enhance Growth Characteristics, Pigments Content, and Antioxidant Capacity in Lettuce, Spinach, Kale, Basil, and Sweet Pepper in a Controlled Environment

**DOI:** 10.3390/plants8040093

**Published:** 2019-04-08

**Authors:** Most Tahera Naznin, Mark Lefsrud, Valerie Gravel, Md Obyedul Kalam Azad

**Affiliations:** 1 Department of Biosystems and Technology, Swedish University of Agricultural Sciences, 103-23053 Alnarp, Sweden; 2 Bioresource Engineering Department, McGill University, Ste-Anne-de-Bellevue, QC H9X3V9, Canada; mark.lefsrud@mcgill.ca; 3 Plant Science Department, McGill University, Ste-Anne-de-Bellevue, QC H9X3V9, Canada; valerie.gravel@mcgill.ca; 4 Department of Bio-Health Technology, College of Biomedical Science, Kangwon National University, Chuncheon 24341, Korea; azadokalam@gmail.com

**Keywords:** blue LED, red LED, lettuce, spinach, basil, kale, pepper, growth, antioxidant, pigment

## Abstract

The aim of this study was to investigate the different combinations of red (R) and blue (B) light emitting diode (LEDs’) lighting effects on growth, pigment content, and antioxidant capacity in lettuce, spinach, kale, basil, and pepper in a growth chamber. The growth chamber was equipped with R and B light percentages based on total light intensity: 83% R + 17% B; 91% R + 9% B; 95% R + 5% B; and control was 100% R. The photosynthetic photon flux density (PPFD), photoperiod, temperature, and relative humidity of the growth chamber were maintained at 200 ± 5 μmol m^−2^ s^−1^, 16 h, 25/21 ± 2.5 °C, and 65 ± 5%, respectively. It is observed that the plant height of lettuce, kale, and pepper was significantly increased under 100% R light, whereas the plant height of spinach and basil did not show any significant difference. The total leaf number of basil and pepper was significantly increased under the treatment of 95% R + 5% B light, while no significant difference was observed for other plant species in the same treatment. Overall, the fresh and dry mass of the studied plants was increased under 91% R + 9% B and 95% R + 5% B light treatment. The significantly higher flower and fruit numbers of pepper were observed under the 95% R + 5% B treatment. The chlorophyll *a*, chlorophyll *b*, and total chlorophyll content of lettuce, spinach, basil, and pepper was significantly increased under the 91% R + 9% B treatment while the chlorophyll content of kale was increased under the 95% R + 5% B light treatment. The total carotenoid content of lettuce and spinach was higher in the 91% R + 9% B treatment whereas the carotenoid content of kale, basil, and pepper was increased under the 83% R + 17% B treatment. The antioxidant capacity of the lettuce, spinach, and kale was increased under the 83% R + 17% B treatment while basil and pepper were increased under the 91% R + 9% B treatment. This result indicates that the addition of B light is essential with R light to enhance growth, pigment content, and antioxidant capacity of the vegetable plant in a controlled environment. Moreover, the percentage of B with R light is plant species dependent.

## 1. Introduction

Light is one of the most important environmental factors for plant growth, development, and plant metabolites’ accumulation [1]. Specific wavelengths of light have precise effects on plants. For example, wavelengths of light in the red (R) and blue (B) region are absorbed by chlorophyll while carotenoids absorb strongly in the B region with a maximum peak occurring at 454 and 448 nm, respectively [2]. Also, B light steers plant growth, leaf expansion, photomorphogenesis, stomatal opening, photosynthesis, and the accumulation of pigments [3]. R light plays an important role in controlling the functions of the chloroplast, stem, and petiole growth as well as the reproductive system [4]. Suitable light spectra of light-emitting diodes (LEDs) is of paramount importance for the horticulture industry. LEDs provide versatile options to control vital plant processes through a tailored light spectrum, which can be fitted with the absorption of plant photoreceptors [5]. Moreover, solid-state LEDs provide many advantages, such as longevity, safety, emitting lower heat, improved quality of light, and reduced electricity consumption [6,7,8].

Synergetic effects of different wavelength were observed when mixtures of R and B LEDs were used to irradiate plants. Combined R and B LEDs enhanced fresh mass and dry mass content of various vegetables compared with R LEDs alone [9,10,11,12]. However, other studies revealed contrasting results, such as a combination of R and B LEDs decreased the fresh and dry mass of lettuce compared with R LEDs alone [13,14,15]. This inconsistent result remains as a grey area, which demands further research.

Mixtures of R and B LEDs increased the accumulation of chlorophyll and antioxidants in lettuce [14,15], antioxidants in basil [16], and carotenoids in pepper [17]. Carotenoid and chlorophyll synthesized by all plants are components of photosynthesis and serve critical functions in plant biology, including light harvesting, quenching of photooxidation, colouring of plants, and providing nutritional benefits as a precursor of essential vitamins and antioxidants for human beings. Those results have shown the influence of an optimized light quality for increasing phytochemical concentrations.

Lettuce (*Lactuca sativa*) is the most cultivated plant in greenhouses worldwide. Spinach (*Spinacea oleracea*) is an increasingly popular plant with consumption steadily increasing globally because of its rich source of nutrients [18,19]. Kale (*Brassica oleracea* var. *sabellica*) has gained popularity as a health promoting plant food [20,21]. Basil (*Ocimum basilicum*) is an aromatic plant and is a popular culinary herb. The potential health benefits of basil include strengthening the immune system, alleviating metabolic disorders, cognitive enhancement as well as benefits to oral and skin health [22]. The popularity of peppers (*Capsicum annuum* L.) for fresh market consumption or ready-to-eat foods has been augmented significantly during the past decades, and are mostly produced in protected environments [23]. R and B wavelengths influence health beneficial phytochemical accumulations [24,25]. It is difficult to understand how plants respond to changes to different percentages of R and B light because most existing studies have revealed inconsistent results.

However, there is very limited study on examining the effects of different percentages of R and B LEDs on plant growth, pigment accumulation, and antioxidant capacity in a wide range of crop varieties at the same time. Therefore, this study aimed at determining the effect of different combinations of R and B LEDs on growth, pigments, and antioxidant capacity in lettuce, kale, spinach, basil, and pepper in a closed production system.

## 2. Materials and Methods

### 2.1. Plant Material and Growth Conditions

These experiments were performed at Macdonald Campus, McGill University, Canada. Seeds of lettuce (Buttercrunch, Lot P172, Stokes seed Ltd., Thorold, ON, Canada), spinach (Unipack 151, Lot 286A, Stokes seed Ltd., Thorold, ON, Canada), basil (G Lemon Basil, Lot 163A, Stokes seed Ltd., Thorold, ON, Canada), kale (Vates Blue Curled, Lot 168A, Stokes seed Ltd., Thorold, ON, Canada), and dwarf sweet pepper (Redstart-dwarf Sweet Pepper, Lot No: 261F, Stokes seed Ltd., Thorold, ON, Canada) were sown into rockwool growing cubes (Grodan A/S, Dk-2640, Hedehusene, Denmark) and germinated in a growth chamber (E15, Conviron, Winnipeg, MB, Canada) under fluorescent light (200 µmol m^−2^ s^−1^). All vegetable seedlings (4–6 cm height) were thinned to one seedling per cube and each cube was placed in trays for hydroponic culture (deep water culture) and transferred to the treatment LED light chamber. Plants were provided with a half strength Hoagland nutrient solution. The pH and electrical conductivity in nutrient solution were measured weekly and adjusted to pH 5.8 and electrical conductivity to 1.85 mS cm^−1^. All leafy vegetables, including lettuce, spinach, basil, and kale, were harvested after five weeks of treatment and sweet pepper was harvested after seven weeks. The experiment was repeated twice.

### 2.2. LED Light Treatments

Plants were illuminated by light emitting diodes (LEDs) with different percentages of red (R, 661 nm) and blue (B, 449 nm) light. Four spectral treatments were used in this study, namely 83% R + 17% B, 91% R + 9% B, 95% R + 5% B, and 100% R (control). The photoperiod was 16/8 h (day/night), photosynthetic photon flux density (PPFD) was 200 ± 5 μmol m^−2^ s^−1^, day/night temperature was 21 °C ± 2.5 °C, and relative humidity was 65 ± 5% in the growth chamber. The LED lights were prototypes from General Electric Lighting Solutions (Lachine, QC, Canada). These consisted of 1.78 m × 0.08 m × 0.02 m linear fixtures, on which were placed an array of 16 LEDs. Irradiance was measured routinely using a quantum sensor (MQ-200; Apogee Instruments, Logan, UT, USA).

### 2.3. Plant Growth and Biomass Measurement

Plant height and the number of leaves from the plants were recorded after five weeks. Plant height was measured to the tip of the youngest leaf. The fresh mass (FM) of the plant parts were determined immediately after harvesting. The drying temperature was 80 °C and duration of drying was no less than 72 h until a stable mass was attained. Plant tissues were weighed again after drying to record dry biomass. Leaf tissue samples and sweet pepper fruits were frozen (−22 °C) before lyophilization. Phytochemicals and antioxidant properties were analysed from freeze dried leaf tissues (leafy vegetables) and fruit tissues (pepper).

### 2.4. Chlorophyll and Carotenoid Analysis

For chlorophyll and carotenoid analysis, the method described by Lichtenthaler [26] was used. Aliquots of 100 mg of dried and ground leaf tissue and sweet pepper fruit tissue were added to 10 mL of 80% acetone, mixed well, and incubated overnight at 4 °C in darkness. The supernatant was withdrawn after centrifugation at 5000 rpm for 10 min at 25 °C and absorbance were recorded at 646.8, 663.2, and 470 nm against 80% acetone blank using a spectrophotometer (PowerWave™ XS Microplate Reader, BioTek Instruments, Inc. Vermont, USA). The amount of chlorophyll a, chlorophyllb, total chlorophyll, and carotenoids were determined according to Lichtenthaler (1987).

### 2.5. Antioxidant Capacity Measurement

Antioxidant activity was measured by the scavenging capacity of the stable 2, 2-diphenyl-1 picryl hydrazyl (DPPH) free radical assays [27,28]. Briefly, 25 µL of appropriately diluted samples were added to 200 µL of DPPH solution (dissolved in methanol) in a well of a 96-well plate. The mixture was allowed to react at room temperature in the dark for 4 h before the absorbance was measured at 517 nm against a methanol blank using the spectrophotometer (PowerWave™ XS Microplate Reader, BioTek Instruments, Inc. Vermont, USA). The radical scavenging activity (RSA) percentage was determined from Equation (1), considering the absorbance of methanol as the control (A_0_) and the sample (A_x_):

RSA (%) = [(A_0_ − A_x_)/A_0_] × 100 (1)

### 2.6. Statistical Analysis

The statistical analysis was performed using analysis of variance (ANOVA). Data analysis was processed by one-way analysis of variance (ANOVA), combined with the Tukey’s (Tukey Simultaneous 95% CIs) least significant difference (LSD) test at the confidence levels of *p* < 0.05 by using Minitab version 17. The results were expressed as mean values and their standard errors (SE) using MS Excel software.

## 3. Results and Discussion

### 3.1. Effect of Different Combinations of Red and Blue Light on the Plant Growth Characteristics

#### 3.1.1. Plant Height and Growth Features

**Figure 1:**
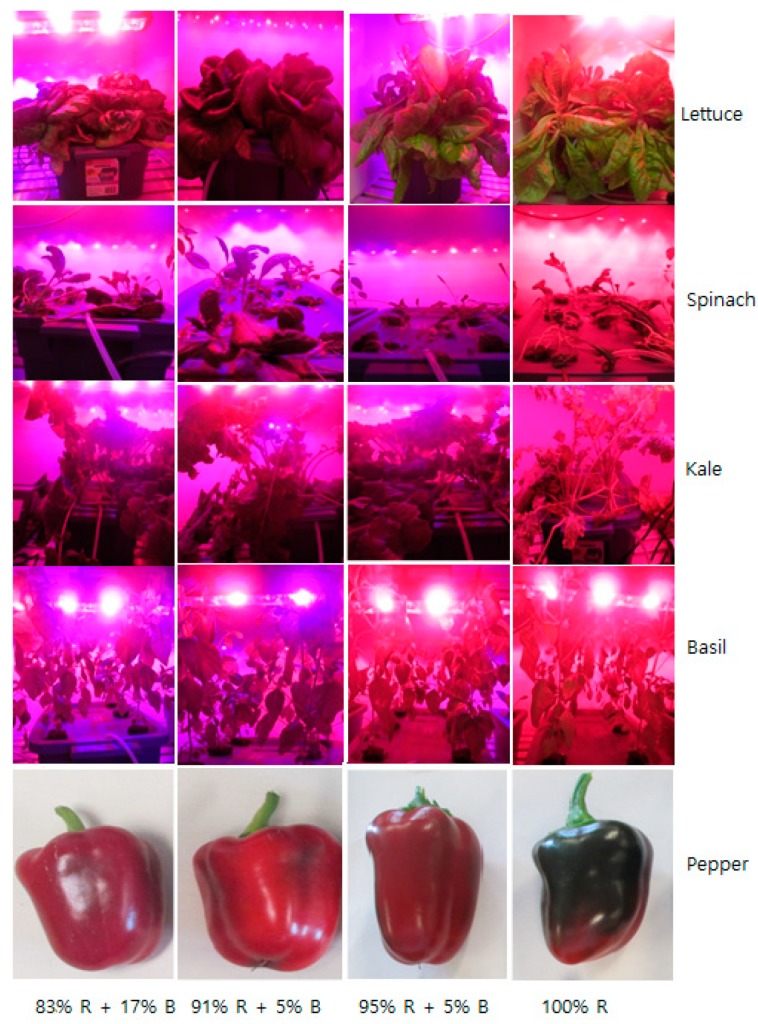
Morphological features of different plant species grown under various ratios of R and B light.

The different percentages of R and B LEDs showed great influence on plant height and growth features (Table 1, Figure 1). Overall, the 100% R light condition increased the plant height of all cultured plants except pepper. The plant height of the pepper plant increased in the 95% R + 5% B light treatment. However, it was shown that the plant height decreased with the increasing percentage of B LEDs in lettuce and kale. The significantly decreased plant height of kale and pepper was observed under the treatment of 83% R + 17% B LEDs, as well as an observed reduced plant height of lettuce under the 95% R + 5% B LEDs. On the contrary, the different percentages of R and B LEDs did not show any significant influence on the plant height of spinach and basil. Regarding the morphological features, all studied plant species had a compact leaf arrangement with dark green colour and small petioles when grown under the 91% R with 9% B light treatment, whereas plants grown under a higher R light fraction had pale green coloured spread leaves with long petioles. It was also observed that the pigmentation of the pepper fruit was not developed under the 100% R light. Moreover, the desired fruit shape and size was obtained under the 91% R + 9% B light treatment (Figure 1).

Light quality regulates plant growth through various photoreceptors, which stimulate signal transduction systems by various mechanisms to change plant morphology [29]. In the present study, the plant height of all plants increased with increasing R light and the highest plant height was observed under the treatment of 100% R LEDs except for spinach and basil. The plant height of spinach and basil did not show any significant difference among the treatments. It has been reported that plants under 100% R light have increased plant height [30,31]. Previously, pepper plants grown under the 100% R light showed greater plant heights than under the 10% B with 90% R light [32]. Similarly, cherry tomato plants grown under 100% R light showed greater plant heights than plants grown under 50% B with 50% R light [33]. The photoreceptor cryptochromes are responsible for the inhibition of plant height and have maximal activity when stimulated with B light [34,35]. In this study, 449 nm was the peak wavelength of the B LEDs, which is within the range of maximal activity of the cryptochrome. Therefore, the inhibition of plant height with the increase of B LEDs is likely attributed to the stimulated cryptochrome photoreceptor.

#### 3.1.2. Leaf Number

It was observed that different percentages of R and B LEDs had an influence on the plant leaf number (Table 1). In spinach and basil, a significantly higher leaf number was observed under the treatment of 91% R with 9% B LEDs than the treatment of 100% R LEDs. In pepper, a significantly higher leaf number was observed under the treatment of 95% R with 5% B LEDs in comparison with 100% R LEDs. In this study, the different percentages of R and B LEDs showed a significant influence on the leaf number of spinach, basil, and pepper, but not lettuce and kale. A previous study showed no differences in the leaf number in cucumber under different ratios of R and B light [36]. The effect of different percentages of R and B LEDs on the leaf number is plant species dependent.

#### 3.1.3. Shoot Fresh and Dry Mass

It is clearly shown that plant shoots’ fresh and dry mass increased with the increase of the B light in the R light combination (Table 1). The fresh and dry mass accumulation of lettuce, spinach, and basil increased with the increase of B light till 9% with the R light.

The 91% R + 9% B treatment showed significantly increased fresh and dry mass in lettuce, spinach, and basil in comparison with the 100% R LEDs treatment. Whereas, the fresh and dry mass accumulation in kale and pepper increased with the increase of B light up to 5% then decreased, and a significantly higher accumulation was observed under the 95% R + 5% B light treatment in comparison with the 100% R LEDs treatment. The fresh mass in lettuce, spinach, and basil was 1.2, 1.4, and 1.4 times higher and the dry mass was 1.7, 1.4, and 1.4 times higher for plants cultured under the 91% R with 9% B treatment, respectively, compared to the 100% R treatment. The accumulation of fresh mass in kale and sweet pepper was 2.2 and 1.5 times higher whereas the dry mass was 1.5 and 1.9 times higher for plants cultured under the 95% R with 5% B treatment, respectively, compared to the treatment 100% R treatment.

A previous study showed that pepper plants grown under 100% R light had a lower plant dry mass compared to plants grown under 99% R with 1% B light [32]. The present study showed decreased fresh and dry mass accumulation for all plants grown under 100% R light in comparison with the R and B light combination. Similarly, chili pepper plants cultured under 100% R light showed significantly lower fresh and dry mass compared to plants grown under RB light [17]. In contrast, tomato, salvia, and petunia had greater shoot fresh mass when grown under 100% R light than under 25% B with 75% R light [37]. Similarly, leaf lettuce showed higher fresh and dry mass accumulation under 100% R light compared to the R with the B light combination [15]. Similarly, cucumber plants cultured under 100% R light had greater shoot fresh mass and dry mass in comparison with the mixture of R and B light [36]. This may indicate that R and B light effects on plant biomass could depend on the species. 

#### 3.1.4. Flower and Fruit of the Pepper Plant

**Figure 2:**
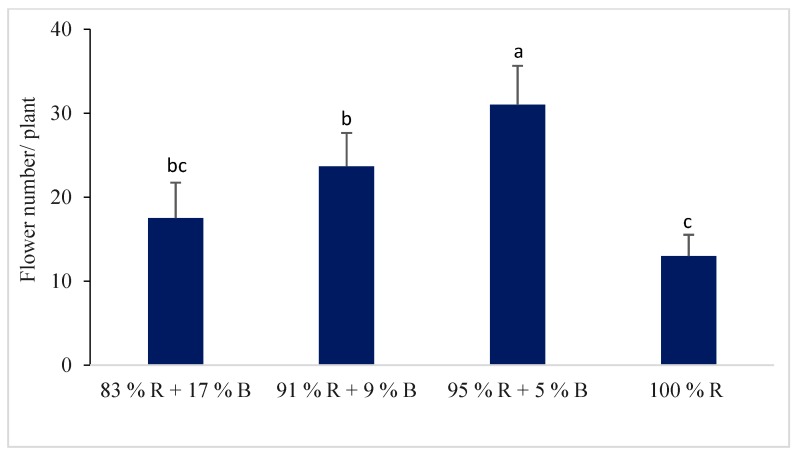
The effect of different percentages of red (R) and blue (B) LEDs on the initiation of the flowering of the sweet pepper. Bars represent the standard deviation (n = 6). Values with different letters are significantly different from each other (*p* < 0.05).

**Figure 3:**
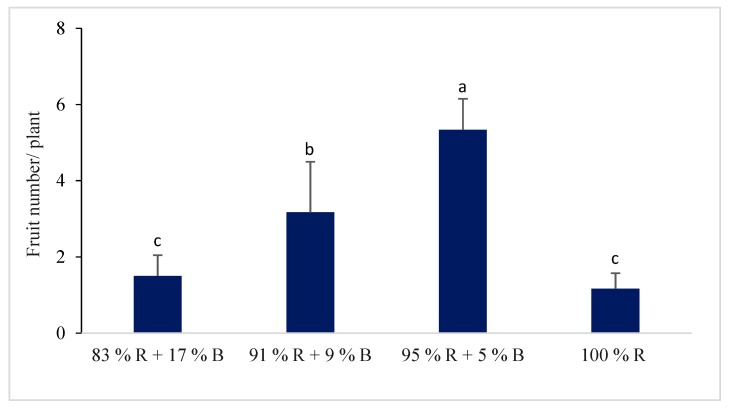
The effect of different percentages of red (R) and blue (B) LEDs on the fruit number of the sweet pepper. Bars represent the standard deviation (n = 6). Values with different letters are significantly different from each other (*p* < 0.05).

Different percentages of R and B LEDs showed a significant impact on the flower and fruit number of the pepper plant (Figure 2 and Figure 3). The flower and fruit numbers of pepper plants were higher under the combinations of different RB light ratios compared to 100% R light. The significantly higher number of flowers and fruits in the pepper plant were observed under the 95% R with 5% B treatment among the different RB light ratios. The fruit number per plant was 4.6 times higher under the 95% R + 5% B treatment whereas the fruit number was 1.3 and 2.7 times higher under the 83% R + 17% B treatment and 91% R + 9% B treatment, respectively.

Our study showed that significantly higher flower and fruit numbers were observed under combinations of RB light in comparison with 100% R light. A previous study showed that pepper plants grown under a combination of R with B LEDs showed greater total yields and fruit numbers in comparison with 100% R light [17]. The higher growth and fresh mass of the pepper plants indicates the plants’ good health and fruiting potentiality, thus increasing the number of flowers and fruits of the plants. This may be the result of the maximum photosynthetic efficiency of plants grown under R and B LED, because the spectral energy distribution of R and B light coincides with that of the pigment absorption [38].

The R and B light combination allowed a higher photosynthetic activity than 100% R light [39,40]. A previous study reported that R light enhanced the starch content by inhibiting the translocation of photosynthates out of the leaves, which may have a negative impact on the number of flowers and fruit production [41].

### 3.2. Effect of Different Combinations of Red and Blue Light on the Accumulation of Plants’ Pigment Content, and Antioxidant Capacity

#### 3.2.1. Chlorophyll (chl) and Carotenoid Content

Plants cultured under a different percentage of R and B LEDs had an impact on pigment accumulation (Table 2). The chl *a*, chl *b* and total chl content increased with increasing of B light (Table 2). In lettuce and basil, the chl *a*, chl *b*, and total chl accumulation increased with the increase of B light in the 91% R + 9% B treatment. Therefore, the increased accumulation of chl *a*, chl *b*, and total chl in lettuce and basil under the 91% R + 9% B treatment was determined in comparison with the 100% R treatment. The total chl accumulation in lettuce and basil was 1.2 and 1.3 times higher when plants were cultured under the 91% R + 9% B treatment, respectively, compared with the 100% R treatment. In spinach, kale, and pepper, the total chl accumulation was 1.3, 1.3, and 1.3 times higher when plants were cultured under the 95% R + 5% B treatment, respectively, compared with the 100% red treatment. 

Different combinations of R and B LEDs had a great influence on the carotenoid accumulation in plants (Table 2). The accumulation of carotenoids in lettuce, spinach, and pepper was significantly increased by the 91% R + 9% B treatment. Whereas, in kale and basil, the accumulation of carotenoids was significantly increased in the 83% R + 17% B treatment. In lettuce, spinach, and pepper, carotenoid accumulation was 1.4, 1.4, and 1.6 times higher when plants were cultured under the 91% R with 9% B treatment, respectively, compared to the 100% R treatment. The carotenoid accumulation in kale and basil was 1.3 and 1.8 times higher when plants were cultured under the 83% R with 17% B treatment, respectively, compared to the 100% R treatment.

The primary effect of B light on chl biosynthesis has been reported in previous studies [9,42]. An earlier study showed that the total chl content increased in the chili pepper under the combination of R and B light compared with monochromatic R or B LEDs [17]. Studies have reported an increase in chl content under B LEDs in lettuce [14,43]. In contrast, other studies have reported a decrease in chl concentration in kale and sprouting broccoli under B LED treatment [21,44]. Hogewoning et al. [9] found that the increase of chl content per leaf of cucumber occurred with the increase of B light up to a 50% B + 50% R treatment. Those findings were supported by the results of the current study. The present study shows that the chl *a*, chl *b*, and total chl accumulation in lettuce and basil increased in the 9% B with 91% R treatment. Whereas, chl *a*, chl *b*, and total chl accumulation in spinach, kale, and pepper was increased by the 5% B with 95% R treatment. This finding indicates that different species respond differently to light quality. To promote the photosynthetic process, both B and R wavelengths are necessary, but the role of each wavelength is not equal. Blue light plays an important role in generating and moving chl in plant leaves [45].

Earlier research reported that high carotenoid contents were found in plants cultivated under R with B light [17,46]. There is a positive relationship between B LED treatments and increased carotenoid concentration in leafy greens [21,44,47]. B light is abundantly absorbed by photosynthetic pigments and is a vital catalyst to increase the chl and carotenoid contents in plants [4,9]. Results from the current study showed that plant species have high chl and carotenoid pigments when grown under higher B:R light ratios. This is consistent with the findings of Li et al. [48] and Son et al. [15]. Ma et al. [49] illustrated that the principal gene activity of the enzyme in chl and carotenoid pigments were stimulated by B light, resulting in higher pigment accumulation. The increased chl and carotenoid contents in higher B light is due to the induction of chl and carotenoid synthesis under RB light [50]. Moreover, the significant increase in the content of carotenoids under the higher B:R light treatment might be due to the cryptochrome action under high B irradiance [51].

In the present study, these findings are consistent with the 91% R + 9% B LED treatment for lettuce, spinach, and sweet pepper, which had the highest total carotenoid accumulation as compared to all other treatments. Whereas, kale and basil had the highest total carotenoid accumulation under the 83% R + 17% B treatment in comparison with all other treatments. This finding showed that different plant species respond differently to the light spectrum. This finding also suggests that the B light percentage stimulate carotenoid accumulation in lettuce, spinach, kale, basil, and pepper.

#### 3.2.2. Antioxidant Capacity

**Figure 4:**
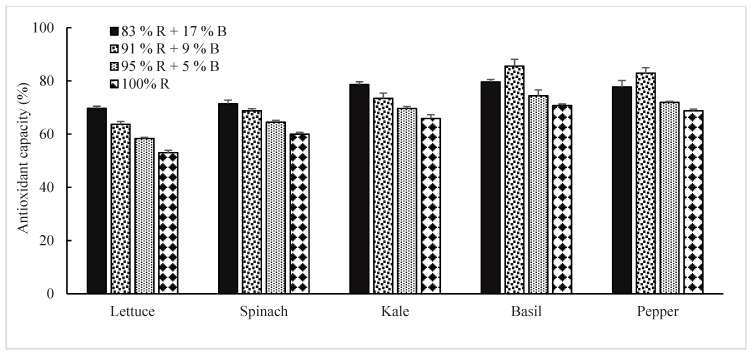
The effect of different percentages of red (R) and blue (B) LEDs on DPPH radical scavenging activity in vegetable crops. Bars represent the standard deviation (n = 6) (*p* < 0.05).

The scavenging capacity of the stable 2, 2-diphenyl-1 picryl hydrazyl (DPPH) free radical scavenging activity in lettuce, spinach, and kale was increased with the increase of B light up to the 83% R + 17% B treatment. Whereas, the free radical scavenging activity in basil and pepper increased with the increase of B light up to the 91% R + 9% B treatment (Figure 4). The antioxidant capacity in lettuce, spinach, and kale was 1.3, 1.2, and 1.2 times higher when plants were cultured under the 83% R with 17% B treatment, respectively, compared to 100% R LEDs. Whereas, DPPH free radical scavenging activity in basil and sweet pepper was 1.2 and 1.1 times higher under the 91% R with 9% B treatment, respectively, compared to the 100% R treatment.

A previous study demonstrated that the combination of R and B LEDs increased the accumulation of antioxidant compounds in plants [12,14]. Son et al. [15] showed that higher portions of B light with R light increased antioxidant activity in the lettuce leaf. Wu et al. [52] reported that a correlation between pigment accumulation (chl and carotenoids) and antioxidant capacity. Liu et al. [53] demonstrated that the activity of light signal transduction *LeHY5* and *LeCOP1LIKE* genes influence light signalling and stimulate pigmentation and nutritional value, which might eventually positively affect the antioxidant capacity of the plant. Yang et al. [54] demonstrated that *LeHY5* and *LeCOP1LIKE* genes are induced by B light mediated signals similar to CRY1. In the current study, the increased antioxidant capacity can be explained on the basis of the pigment content under the higher B light treatment. These findings also suggest that a combination of R and B LEDs plays a vital role in stimulating antioxidant accumulation in vegetable crops.

## 4. Conclusions

In conclusion, the 100% R LED treatment was effective in stimulating plant height in lettuce, kale, and pepper, but not in spinach and basil; however, the 100% R LED treatment had a negative impact on the biomass, chlorophyll content, carotenoid content, and antioxidant levels in lettuce, spinach, kale, basil, and pepper. B mixed with R light, ranging from 5% to 9%, enhanced biomass and total chlorophyll accumulation in lettuce, spinach, kale, basil, and pepper. The B light addition ranging from 9% to 17% with R LEDs stimulated carotenoid contents and antioxidant levels. Our results demonstrate that an appropriate percentage of R and B LEDs can be used to enhance growth, pigment contents, and antioxidant activity in vegetables.

## Figures and Tables

**Table 1:** Plant response of selected vegetable crops to a different percentage of R and B LEDs.

Plants	Treatments	Plant Height (cm)	Leaf No/Plant	Fresh Mass/Plant (g)	Dry Mass/Plant (g)
Lettuce	83% R + 17% B	19.52 ± 2.98 ab	30.33 ± 5.54 a	70.85 ± 5.83 ab	10.45 ± 1.14 ab
	91% R + 9% B	19.33 ± 4.23 ab	26.67 ± 3.27 a	78.45 ± 10.21 a	12.28 ± 3.52 a
	95% R + 5% B	18.17 ± 4.62 b	25.50 ± 3.01 a	72.52 ± 7.80 ab	9.15 ± 1.05 a
	100% R	24.33 ± 2.50 a	24.67 ± 4.13 a	64.82 ± 8.05 b	6.95 ± 0.85 b
	*LSD < 0.05*	*3.68*	*4.11*	*8.12*	1.97
Spinach	83% R + 17% B	31.35 ± 3.59 a	66.67 ± 9.71 a	63.73 ± 6.36 ab	9.88 ± 1.09 ab
	91% R + 9% B	35.6 ± 3.32 a	72.5 ± 8.06 a	68.73 ± 6.87 a	12.13 ± 2.7 a
	95% R + 5% B	29.83 ± 2.93 a	69.17 ± 5.63 a	65.25 ± 3.43 a	11.35 ± 0.50 ab
	100% R	35.17 ± 6.88 a	36.33 ± 9.09 b	47.7 ± 12.09 b	8.45 ± 2.9 b
	*LSD < 0.05*	*4.47*	*8.27*	*7.84*	*2.01*
Kale	83% R + 17% B	46.05 ± 1.79 b	27.83 ± 2.92 a	109.78 ± 9.99 b	26.21 ± 3.34 a
	91% R + 9% B	46.43 ± 2.82 b	28.83 ± 2.04 a	185.6 ± 6.56 a	28.11 ± 4.12 a
	95% R + 5% B	47.38 ± 1.51 ab	28.17 ± 2.14 a	189.95 ± 7.32 a	29.71 ± 3.24 a
	100% R	51.63 ± 4.95 a	29.17 ± 1.94 a	85.45 ± 9.98 c	19.76 ± 3.67 b
	*LSD < 0.05*	*3.08*	*2.29*	*8.61*	*3.61*
Basil	83% R + 17% B	45.68 ± 3.99 a	101.83 ± 2.78 ab	41.2 ± 8.82 ab	10.13 ± 0.73 ab
	91% R + 9% B	47.33 ± 4.34 a	107.5 ± 9.20 a	44.23 ± 3.58 a	13.45 ± 3.22 a
	95% R + 5% B	48.05 ± 5.76 a	103.67 ± 7.34 a	42.92 ± 3.22 ab	11.95 ± 3.54 ab
	100% R	51.12 ± 4.48 a	90.5 ± 10.42 b	32.68 ± 9.63 b	9.37 ± 1.19 b
	*LSD < 0.05*	*4.69*	*7.98*	*6.96*	*2.49*
Pepper	83% R + 17% B	467.48 ± 6.6 c	183.39 ± 8.4 b	650.88 ± 8.8 c	218.37 ± 4.5 b
	91% R + 9% B	492.27 ± 6.1 b	191.61 ± 6.5 b	683.89 ± 7.5 b	305.49 ± 4.9 a
	95% R + 5% B	568.33 ± 6.7 a	215.37 ± 3.6 a	783.71 ± 6.1 a	197.03 ± 4.5 c
	100% R	459.42 ± 4.7 c	161.39 ± 5.0 c	620.82 ± 7.2 d	183.67 ± 4.9 d
	*LSD < 0.05*	*6.1*	*6.1*	*7.5*	*4.7*

Mean separation within columns by the DMRT test at the 5% significant level. (n = 6). Values labelled with different letters in a column are significantly different (*p* < 0.05). *p* ≤ 0.05 different from control (100% R) using Tukey’s (Tukey Simultaneous 95% CIs) least significant difference (LSD) test.

**Table 2:** The effect of different percentages of red (R) and blue (B) LEDs on chlorophyll a, chlorophyll b, total chlorophyll, and carotenoid content in vegetable crops.

Plants	Treatments	chl *a* (µg g^−1^ DM)	chl *b* (µg g^−1^ DM)	Total chl (µg g^−1^ DM)	Carotenoids (µg g^−1^ DM)
Lettuce	83% R + 17% B	555.69 ± 6.4 b	177.46 ± 6.2 c	733.14 ± 8.1 b	201.96 ± 4.8 a
	91% R + 9% B	600.51 ± 5.8 a	199.88 ± 6.8 a	800.39 ± 9.6 a	208.02 ± 5.6 a
	95% R + 5% B	554.67 ± 4.62 b	188.86 ± 5.9 b	743.53 ± 9.8 b	181.85 ± 3.9 b
	100% R	515.89 ± 3.9	145.48 ± 6.1 d	661.38 ± 3.9 c	151.72 ± 7.5 c
	*LSD < 0.05*	*5.3*	*6.2*	*8.2*	5.5
Spinach	83% R + 17% B	566.61 ± 5.1 b	164.46 ± 6.2 b	730.14 ± 8.1 b	221.96 ± 4.8 a
	91% R + 9% B	589.35 ± 7.6 a	188.88 ± 6.8 a	777.39 ± 9.6 a	219.96 ± 4.8 a
	95% R + 5% B	597.16 ± 7.8 a	162.86 ± 5.9 b	759.53 ± 9.8 b	171.85 ± 3.9 b
	100% R	465.68 ± 6.9 c	137.48 ± 6.1 d	602.38 ± 3.9 c	163.72 ± 7.5 c
	*LSD < 0.05*	6.9	*6.2*	*8.2*	5.5
Kale	83% R + 17% B	578.39 ± 8.3 c	203.37 ±7.2 c	781.77 ± 9.2 c	293.13 ± 6.5 a
	91% R + 9% B	671.45 ± 4.9 b	226.65 ±8.0 b	898.09 ± 7.1 b	271.16 ± 5.4 b
	95% R + 5% B	702.74 ± 7.6 a	265.65 ±7.1 a	968.39 ± 8.8 a	261.23 ± 4.9 b
	100% R	556.13 ± 4.2 d	201.13 ±5.6 c	757.26 ± 5.4 d	223.28 ± 7.9 c
	*LSD < 0.05*	*6.5*	*7.0*	*7.8*	*6.3*
Basil	83% R + 17% B	573.27 ± 5.1 c	190.06 ± 4.1 b	763.34 ± 5.3 c	316.67 ± 9.1 a
	91% R + 9% B	644.42 ± 5.6 a	215.35 ± 6.7 a	859.77 ± 3.3 a	295.92 ± 3.9 b
	95% R + 5% B	618.73 ± 7.1 b	201.51 ± 10.7 b	820.24 ± 8.3 b	269.96 ± 6.1 c
	100% R	524.52 ± 5.4 d	162.32 ± 5.3 c	686.85 ± 9.3 d	171.42 ± 8.1 d
	*LSD < 0.05*	*5.8*	*7.1*	*6.9*	*7.3*
Pepper	83% R + 17% B	573.27 ± 5.1 c	190.06 ± 4.1 b	763.34 ± 5.3 c	316.67 ± 9.1 a
	91% R + 9% B	644.42 ± 5.6 a	215.35 ± 6.7 a	859.77 ± 3.3 a	295.92 ± 3.9 b
	95% R + 5% B	618.73 ± 7.1 b	201.51 ± 10.7 b	820.24 ± 8.3 b	269.96 ± 6.1 c
	100% R	524.52 ± 5.4 d	162.32 ± 5.3 c	686.85 ± 9.3 d	171.42 ± 8.1 d
	*LSD < 0.05*	*5.8*	*7.1*	*6.9*	*7.3*

Mean separation within columns by the DMRT test at the 5% significant level. (n = 6). Values labelled with different letters in a column are significantly different (*p* < 0.05). *p* ≤ 0.05 different from control (100% R) using Tukey’s (Tukey Simultaneous 95% CIs) least significant difference (LSD) test.
